# Biomimetic Catalysts Based on Au@ZnO–Graphene Composites for the Generation of Hydrogen by Water Splitting

**DOI:** 10.3390/biomimetics5030039

**Published:** 2020-08-21

**Authors:** Abniel Machín, Juan C. Arango, Kenneth Fontánez, María Cotto, José Duconge, Loraine Soto-Vázquez, Edgar Resto, Florian Ion Tiberiu Petrescu, Carmen Morant, Francisco Márquez

**Affiliations:** 1 Arecibo Observatory, Universidad Ana G. Méndez-Cupey Campus, San Juan, PR 00926, USA; 2 Nanomaterials Research Group, School of Natural Sciences and Technology, Universidad Ana G. Méndez-Gurabo Campus, Gurabo, PR 00778, USA; jcarangolozano@hotmail.com (J.C.A.); kenneth.fontanez@gmail.com (K.F.); mcotto48@uagm.edu (M.C.); jduconge@uagm.edu (J.D.); fmarquez@uagm.edu (F.M.); 3 Materials Characterization Center Inc., Molecular Sciences Research Center, University of Puerto Rico, San Juan, PR 00926, USA; sotol6@uagm.edu (L.S.-V.); edgar.resto@upr.edu (E.R.); 4 IFToMM-ARoTMM, Bucharest Polytechnic University, 060042 Bucharest, Romania; tiberiuflorianion@gmail.com; 5 Department of Applied Physics, Autonomous University of Madrid, 28041 Madrid, Spain; c.morant@uam.es

**Keywords:** hydrogen production, ZnO, gold nanoparticles, graphene, water splitting

## Abstract

For some decades, the scientific community has been looking for alternatives to the use of fossil fuels that allow for the planet’s sustainable and environmentally-friendly development. To do this, attempts have been made to mimic some processes that occur in nature, among which the photosystem-II stands out, which allows water splitting operating with different steps to generate oxygen and hydrogen. This research presents promising results using synthetic catalysts, which try to simulate some natural processes, and which are based on Au@ZnO–graphene compounds. These catalysts were prepared by incorporating different amounts of gold nanoparticles (1 wt.%, 3 wt.%, 5 wt.%, 10 wt.%) and graphene (1 wt.%) on the surface of synthesized zinc oxide nanowires (ZnO NWs), and zinc oxide nanoparticles (ZnO NPs), along with a commercial form (commercial ZnO) for comparison purposes. The highest amount of hydrogen (1127 μmol/hg) was reported by ZnO NWs with a gold and graphene loadings of 10 wt.% and 1 wt.%, respectively, under irradiation at 400 nm. Quantities of 759 μmol/hg and 709 μmol/hg were obtained with catalysts based on ZnO NPs and commercial ZnO, respectively. The photocatalytic activity of all composites increased with respect to the bare semiconductors, being 2.5 times higher in ZnO NWs, 8.8 times higher for ZnO NPs, and 7.5 times higher for commercial ZnO. The high photocatalytic activity of the catalysts is attributed, mainly, to the synergism between the different amount of gold and graphene incorporated, and the surface area of the composites.

## 1. Introduction

If you were asking to identify one of the many challenges that our world is facing right now, you would probably say climate change, energy production, or sustainability. Most of these problems are directly related to the continuous growth of the world population and the use of fossil fuels as our primary energy source [1].

In the case of finding a clean and renewable energy source, multiple candidates have been proposed over the years. One of them is hydrogen. Some of the properties that make hydrogen a good candidate to replace fossil fuels are its abundance, high energy yield, storage capability, and environmental compatibility [2]. Hydrogen is the most abundant element in the universe and can be found in water and biomass. If compared to hydrocarbons, hydrogen can produce 2.75 times more energy, can be stored as a solid, liquid, or gas, and if it is combined with oxygen, no harmful and toxic gases such as nitrogen oxides (NO_x_) or sulfur oxides (SO_x_) are release to the atmosphere [2].

Unfortunately, the vast majority of hydrogen that is produced in the world, comes from a process known as natural gas reforming [3]. As the name says, this process uses natural gas, methane (CH_4_), as the source to obtain hydrogen. This process releases carbon dioxide (CO_2_) to the atmosphere, increasing the amount of this greenhouse gas and exacerbating global warming [3].

Photosynthesis offers an excellent model for designing an artificial solar energy conversion system for clean fuel generation. In nature, electrons are provided to the reaction center of the photosystem-II in four consecutive proton coupled electron transfer steps, and ultimately appear as reduced carbon derived products that form the basis of biological activity. Inspired by natural principles, for decades there has been a continuous effort to design artificial photosynthetic assemblies based on the use of solar energy to generate oxygen and hydrogen by water splitting [4,5,6]. Over the years, multiple candidates such as titanium dioxide (TiO_2_), zinc oxide (ZnO), tungsten trioxide (WO_3_), cadmium sulfide (CdS), among others [7,8,9,10,11] have been used to produce hydrogen via water splitting [12,13]. From all these photocatalysts, TiO_2_ has been extensively studied over the years mainly due to its chemical stability, abundance, non-toxicity, and high hydrogen yield [12].

Similar to titanium oxide, ZnO has also been demonstrated to be chemically stable, easy to produce, non-toxic, abundant, and environmentally-friendly [9,10], although unlike titanium dioxide, ZnO has been widely used for the degradation of organic pollutants and energy storage [14,15,16]. Some authors [17,18] consider that ZnO shows some disadvantages for the production of hydrogen by water splitting, especially the recombination of photogenerated electron–hole pairs, fast backward reaction, and the inability to use visible light. To try to solve these limitations, different approaches have been implemented over the years. One of them has been the incorporation of noble metal to the surface of the catalysts [17,18]. Among noble metals, gold has gained much attention since the 1980s because of its wide range of applications, including electronics, photodynamic therapy, delivery of therapeutic agents, sensors, probes, diagnostics, and catalysis [19,20]. Multiple pathways to incorporate gold nanoparticles (Au NPs) can be found in the literature. Methods such as coprecipitation [21], chemical reduction [22], phytochemical reduction [23], and the hydrothermal approach [24] have been successfully implemented over the years. All these synthesis procedures take into account parameters such as the preparation procedure, gold loading (percentage of gold weight on the material), particle size, dispersion (percentage of support surface covered by gold), and shape of the particles.

Recently, another approach that has drawn a lot of attention is the use of graphene as a co-catalyst for the production of hydrogen via water splitting. Graphene has unique properties such as high thermal conductivity, excellent mobility of charge carriers, large surface area, and good mechanical stability [25]. As a co-catalyst, graphene has significant advantages, including that it (i) provides a support for anchoring well-dispersed metallic or oxide nanoparticles; (ii) works as a highly conductive matrix for enabling good contact throughout the matrix; (iii) induces an easy electron transfer from the conduction band of the semiconductor to graphene because of the large energy level offset formed at the interface, leading to an efficient charge separation; and (iv) acts as an efficient co-catalyst for H_2_ evolution because of its large specific surface area and superior electron mobility [26].

There are several approaches reported on the literature to prepare graphene–ZnO composites. For example, Tien and group [27] used a microwave-assisted solvothermal process, whereas Ong and group [28] reported the preparation of the composites using a chemical deposition–calcination approach.

There is very limited information in the literature on the production of hydrogen via water splitting by combining graphene and ZnO. Haldorai and Shim [29] reported the production of hydrogen via water splitting by employing a supercritical fluid mediated synthesis. They reported that the composites exhibited enhanced photocatalytic activity, because the ZnO particles on the graphene sheets captured light energy and acted as electron mediators.

To our knowledge, no results have been reported on the incorporation of graphene and gold nanoparticles on the surface of ZnO for the production of hydrogen by water splitting. The information found in the literature is very limited and focuses on the degradation of dyes and nitrobenzene under visible and UV light. For example, Zeng et al. [30] and Wang et al. [31] reported high photocatalytic activity for the degradation of rhodamine B and methylene blue, respectively. They found that the combination of gold and graphene on the surface of ZnO allows the system to use visible and UV light, and more importantly, greatly improves the degradation percentage compared to pure ZnO and Au–ZnO. On the other hand, Roy et al. [32] reported on the efficient reduction of nitrobenzene under UV and visible light, in which the catalyst reduced 97.8% of the original compound.

Due to the lack of information on the production of hydrogen by water splitting using Au@ZnO–graphene composites, the objectives of this research focused mainly on (i) synthesizing ZnO with different morphologies (nanoparticles and nanowires); (ii) incorporating different amounts of gold nanoparticles (1 wt.%, 3 wt.%, 5 wt.%, and 10 wt.%) and graphene (1 wt.%) to the surface of the as-synthesized catalysts and to its commercial form; and (iii) characterizing the photocatalytic activity of the compounds by studying the production of hydrogen by water splitting under UV–vis radiation. Au@ZnO–graphene-based catalysts were characterized using HRTEM, UV–vis spectroscopy, BET surface area, XRD, XPS, Raman spectroscopy, and GC–TCD.

## 2. Materials and Methods

### 2.1. Reagents

All reagents were used as received and all the solutions were prepared using deionized water (Milli-Q water, 18.2 MΩ cm^−1^ at 25 °C). Zn(CH_3_COO)_2_ • 2H_2_O (98+%, ACS Reagent), HAuCl_4_•3H_2_O (ACS Reagent, 49.0+% Au basis), ethanol (95%), and NaBH_4_ (+99.9%) were provided by Sigma Aldrich (Milwaukee, Wisconsin USA). NaOH (98+%) and ZnO (99.99%) were acquired from Alfa Aesar (Ward Hill, Massachusetts USA). Graphene (99%) was provided by Cheap-Tubes (Grafton, Vermont USA). For photocatalytic experiments, Na_2_S (99.9+%) and Na_2_SO_3_ (98+%) were obtained from Sigma Aldrich (Milwaukee, Wisconsin USA) and used as sacrificial reagents.

### 2.2. Synthesis of Nanomaterials

ZnO nanowires (ZnO NWs) were obtained according the method described elsewhere [33]. Zinc oxide nanoparticles (ZnO NPs) were synthesized following the method used by Nejati et al. [34]. The deposition of Au NPs and graphene on the surface of ZnO NWs, ZnO NPs, and the commercial ZnO (commercial ZnO) was based on the method described by Naldoni et al. [35], later modified by Wang et al. [15]. In a typical synthesis, 200 mg of the product (ZnO NWs, ZnO NPs, and commercial ZnO) containing the gold nanoparticles was dispersed in a solution containing 10 mL of ethanol and 40 mL of deionized water, and the mixture was vigorously stirred for 30 min. Subsequently, 2 mg of graphene was added, and the suspension was kept under stirring for 1 h. After that, the product was collected and centrifuged 3 times with deionized water and dried overnight to 60 °C. Finally, the product was collected, sealed, and stored at room temperature. The different Au@ZnO–graphene composites were identified as *x*%Au@ZnO–graphene. The numbers (*x*%) correspond to the weight percentage of Au NPs in the sample. In all cases, the amount of graphene was 1 wt.%.

### 2.3. Characterization of the Catalysts

The catalysts were characterized by high resolution transmission electron microscopy (HRTEM), using a JEOL 3000F. XPS measurements were performed on an ESCALAB 220i-XL spectrometer, using the non-monochromated Mg Kα (1253.6 eV) radiation of a twin-anode, operating at 20 mA and 12 kV in the constant analyzer energy mode, with a PE of 40 eV. Brunauer Emmett Teller (BET) specific areas were measured using a Micromeritics ASAP 2020, according to N_2_ adsorption isotherms at 77 K. Raman (DXR Thermo Raman Microscope, employing a 532 nm laser source at 5 mW power and a nominal resolution of 5 cm^−1^) and X-ray diffraction (Bruker D8 Discover X-ray diffractometer, operating at 40 kV and 40 mA in the range of 30–75° at 1° min^−1^) were also used. UV–vis spectroscopy (Shimadzu UV-2401PC) was used as a complementary technique to determine the absorption edge of the catalysts.

### 2.4. Photocatalytic Experiments

The production of hydrogen via water splitting was measured by adding 50 mg of the *x*%Au@ZnO–graphene catalyst into 100 mL of deionized water and transferring this suspension to a 250 mL quartz reactor. Then, solutions of 0.02 M Na_2_SO_3_ and 0.4 M Na_2_S were added as sacrificial reagents. After that, the solution was thermostatized at 20 °C and purged for 30 min with nitrogen (N_2_). Finally, the reaction mixture was irradiated with UV–vis light for 2 h using different filters to select the appropriate wavelength (280 nm, 320 nm, 400 nm, and 500 nm). The produced hydrogen was quantified by gas chromatography (GC), using a thermal conductivity detector (GC–TCD, Perkin-Elmer Clarus 600) [18].

## 3. Results and Discussion

### 3.1. Characterization of Catalysts

**Figure 1:**
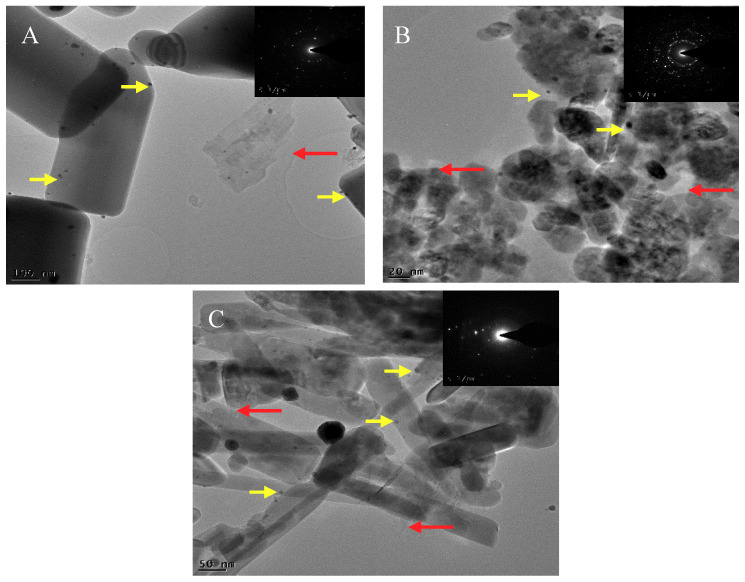
High resolution transmittance electron microscopy (HRTEM) images of 10%Au@commercial ZnO–graphene (**A**), 10%Au@ZnO NPs–graphene (**B**), and 10%Au@ZnO NWs–graphene (**C**). The red and yellow arrows indicate the presence of graphene and gold, respectively, and the insets correspond to the selected area of electron diffraction (SAED) patterns.

The characterization of the different ZnO supports and Au@ZnO-based catalysts was shown in our previous research [18]. On these catalysts, 1 wt.% graphene was incorporated. Figure 1 shows the HRTEM images and the selected area electron diffraction (SAED) patterns of the 10%Au@commercial ZnO–graphene (Figure 1A), 10%Au@ZnO NPs–graphene (Figure 1B), and 10%Au@ZnO NWs–graphene (Figure 1C) composites. The 10%Au@commercial ZnO–graphene composite consisted of non-homogenous particles with different sizes (lengths and diameters greater than 50 nm) and shapes. Homogeneous spherical gold nanoparticles, with diameters of less than ca. 10 nm, were distributed on the surface of the catalyst. Graphene sheets of different sizes were also distributed unevenly through the sample. According to Wang et al. [15], it is believed that close and homogeneous contact between Au, support, and graphene favors the transfer of photogenerated electrons between them, thus improving charge separation and photocatalytic efficiency. As in the case of the commercial catalyst, the 10%ZnO NPs–graphene catalyst showed non-homogeneous particles of different sizes and shapes, with lengths and diameters greater than 50 nm. The non-homogeneous gold nanoparticles were unevenly distributed throughout the sample, presenting a spherical morphology with diameters of less than 10 nm. Graphene was also unevenly distributed throughout the sample and served as a support for ZnO particles and gold nanoparticles. In the case of the 10%Au@ZnO NWs–graphene catalyst, the incorporation of graphene and gold considerably modified the pristine material. The catalyst consisted of non-homogeneous wires, with an estimated length greater than 300 nm and diameters above 50 nm. Gold nanoparticles, with spherical morphology and diameters of less than 10 nm, were distributed throughout the sample. Graphene also appeared to be unevenly distributed in the sample, but had intimate contact with the gold nanoparticles and the support. SAED patterns of synthesized gold–graphene-based composites were characteristic of monocrystalline materials.

Table 1 shows the BET surface area results of the different Au@ZnO–graphene composites. The incorporation of the different amounts of Au NPs (1 wt.%, 3 wt.%, 5 wt.%, and 10 wt.%), along with graphene (1 wt.%), increased the surface area of all the catalysts when compared to the unmodified supports [18]. This enhancement suggests an intimate contact between the incorporated materials and the support [35]. Graphene, as explained above, has a very high surface area (~2000 m^2^ g^−1^), which can contribute to increasing the surface area of composites. However, since the amount of graphene, when compared to gold, was minimum, the enhancement of the surface areas of the catalysts could be primarily attributed to the Au NPs. The highest surface area of the commercial support was measured to be 65 m^2^ g^−1^ and was obtained with the 10%Au@ commercial ZnO–graphene. This represents an increase of 47 m^2^ g^−1^ if compared to the unmodified commercial ZnO support. The highest surface area of the Au@ZnO NPs–graphene composites was 117 m^2^ g^−1^ and was measured in the 10%Au@ZnO NPs–graphene catalyst, showing a difference of 50 m^2^ g^−1^ if compared to the result obtained with the unmodified ZnO NPs. For the Au@ZnO NWs–graphene composites, the highest surface area was 247 m^2^ g^−1^, obtained by the 10% Au@ZnO NWs–graphene catalyst. This represents a difference of 80 m^2^ g^−1^ if compared to the surface area of the unmodified ZnO NWs (167 m^2^ g^−1^).

**Figure 2:**
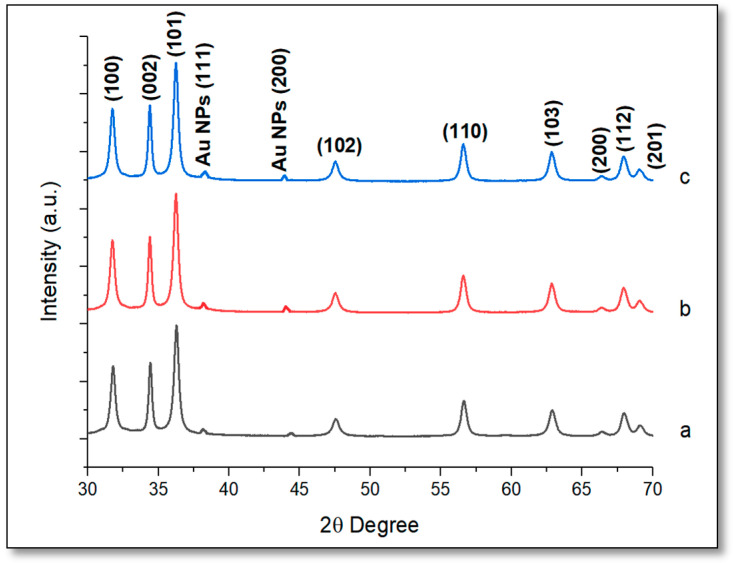
X-ray diffractometer (XRD) diffraction patterns for 10% Au@commercial ZnO–graphene (**a**); 10%Au@ZnO NPs–graphene (**b**); and 10%Au@ZnO NWs–graphene (**c**).

The XRD patterns of the different composites with a gold loading of 10 wt.% are shown in Figure 2. The characteristic peaks of wurtzite crystalline phase (ca. 32.0° (100), 34.8° (002), 36.0° (101), 47.5° (102), 56.2° (110), 62.8° (103), 66.0° (200), 67.5° (112), 68.8° (201)) were observed in all the catalysts [16]. In all cases, reflections at 38.2° and 44.4° were observed, which have been associated with Au (111) and (200), respectively [20], indicating that Au^3+^ had been reduced to Au^0^, with the usual fcc structure. Au NPs showed other characteristic peaks of lower intensity at 64.7° (220) and 77.8° (311), which could not be identified in the catalysts. Applying the Scherrer formula [36], an estimate of the mean size of the gold nanoparticles could be made, providing a value of ca. 15 nm in all cases. This value is very close to that determined by HRTEM (<20 nm). Graphene, on the other hand, has characteristic peaks at ca. 24.5° and 44.2° [24]. These peaks were not identified in any of the composites. The amount of graphene incorporated to the Au@ZnO–graphene composites was very low when compared to the amount of the support and gold loading, and it is possible that the signal emitted by graphene was very weak and not able to be detected by the instrument.

**Figure 3:**
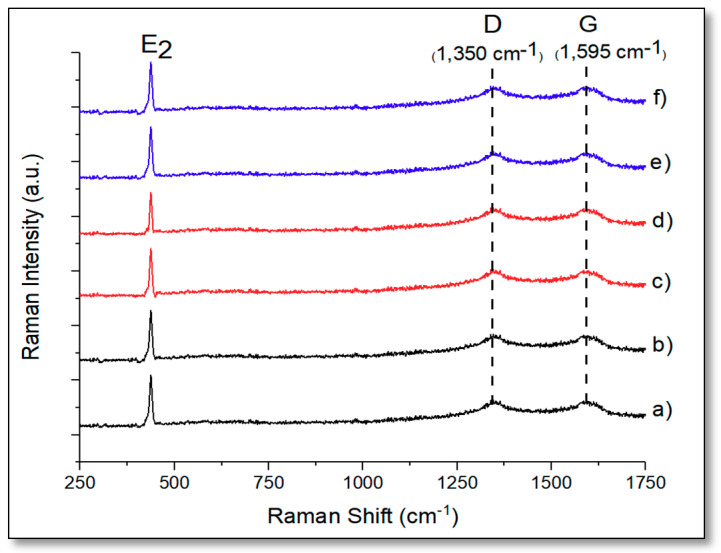
Raman spectra of 5%Au@commercial ZnO–graphene (**a**); 10%Au@commercial ZnO–graphene (**b**); 5%Au@ZnO NPs–graphene (**c**); 10%Au@ZnO NPs–graphene (**d**); 5%Au@ZnO NWs–graphene (**e**); and 10%Au@ZnO NWs–graphene (**f**).

The Au@ZnO–graphene composites containing gold loadings of 5 wt.% and 10 wt.% were characterized by Raman spectroscopy (see Figure 3). Graphene has two characteristics peaks at ca. 1350.0 cm^−1^ and 1595.0 cm^−1^, known as D- and G-bands, respectively [37]. The D-band (1350 cm^−1^) has been related to the defects and structural disorder in graphene sheets, whereas the G-band (1595 cm^−1^) has been ascribed to the stretching of the sp^2^ hybridized carbon–carbon bonds [37]. These two bands were observed in all the gold–graphene composites, including those with gold loadings of 1 wt.% and 3 wt.%. The ratio of the intensity between the D- and G-band is a measure of the degree of disorder in graphene [38]. The narrow strong band at ca. 437.0 cm^−1^ (*E*_2_ modes) is present in all the composites and it has been ascribed to motion of Zn in the wurtzite phase [16]. No gold bands were found for any of the composites.

**Figure 4:**
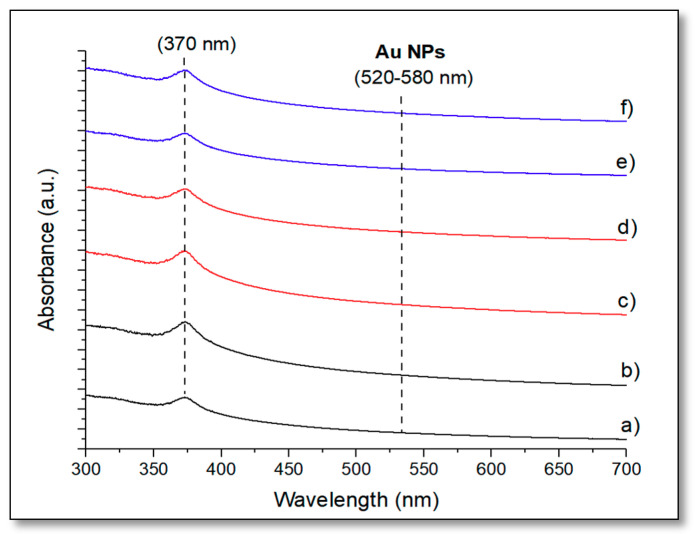
Ultraviolet–visible light (UV–vis) spectra of different composites: 5%Au@commercial ZnO–graphene (**a**); 10%Au@commercial ZnO–graphene (**b**); 5%Au@ZnO NPs–graphene (**c**); 10%Au@ZnO NPs–graphene (**d**); 5%Au@ZnO NWs–graphene (**e**); and 10%Au@ZnO NWs–graphene (**f**).

Au@ZnO–graphene composites, with gold loadings of 5 wt.% and 10 wt.%, were also characterized by UV–vis spectroscopy (Figure 4). All the catalysts presented a similar absorption range between 325 nm and 400 nm, showing a maximum at ca. 370 nm. Interestingly, despite the introduction of graphene and gold, all the composites had almost the same absorption edge as the unmodified catalysts, indicating that there was a consistent band gap of nanocrystalline ZnO within the Au@ZnO–graphene composites. This suggests that no carbon species were incorporated into the lattice of ZnO. Because the impurity level would have shifted the absorption edge to a higher wavelength [39]. No gold peaks (~520–580 nm) were detected for any of the gold loadings incorporated. This might be attributed to the high dispersity of the Au NPs through the samples.

**Figure 5:**
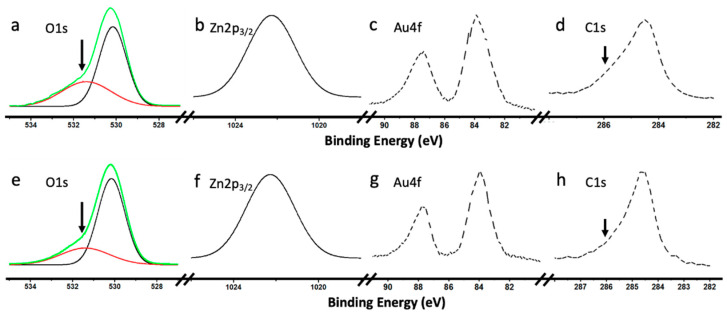
X-ray photoelectron spectroscopy (XPS) spectra of Zn 2p_3/2_, O 1s, Au 4f, and C1s taken from as-grown Au@ZnO NWs–graphene (**a**–**d**), and Au@ZnO NPs–graphene (**e**–**h**).

The catalysts were also characterized by XPS. Figure 5 shows the most relevant spectra of 10%Au@ZnO NWs–graphene and 10%Au@ZnO NPs–graphene. In both systems (Figure 5a,e), the O1s showed a main peak at ca. 530.2 eV, which was assigned to O^2−^ ions in the Zn–O bonds, and a shoulder around 531.5 eV, assigned to O^2−^ ions in the oxygen deficient regions, respectively [18]. As observed, the contribution of this secondary peak was clearly greater in ZnO NWs than in ZnO NPs. As it will be shown later, the highest reactivity was observed in catalysts based on ZnO NWs, so this behavior could be justified thanks to the existence of crystalline defects, as already described in previous works [18]. In fact, surface defects in crystalline ZnO affect its electrical properties, increasing electrical conductivity, which undoubtedly could have positive effects on photocatalysis with these materials. In both catalysts, the Zn2p_3/2_ spectra showed a single component that was unambiguously assigned to Zn^2+^ in ZnO (see Figure 5b,f). The presence of metallic gold (Au^0^) was evidenced by the presence of a doublet in the emission peak at ca. 84.0 eV (4f_7/2_) and 87.7 eV (4f_5/2_) (Figure 5c,g) [40]. No components were observed that could show the presence of Au^3+^, coming from the precursor (HAuCl_4_ • 3H_2_O), which evidenced the complete reduction of gold. Figure 5d,h shows the transition corresponding to C1s. The main peak observed at ca. 284.6 eV was assigned to the carbon backbone of aliphatic/aromatic (sp^3^/sp^2^) carbons, while the component indicated by an arrow, around 286.0 eV could be attributed to carbon in C–O and C–O–C groups [41,42], and to contamination by adsorption of oxidized species (CO, CO_2_).

### 3.2. Photocatalytic Hydrogen Production Via Water Splitting

**Figure 6:**
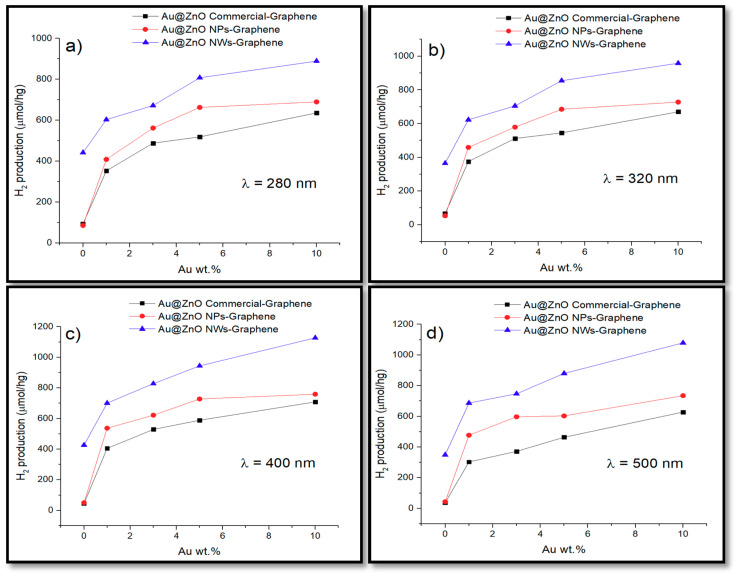
Photocatalytic hydrogen production of the different Au@ZnO–graphene catalysts under irradiation at 280 nm (**a**), 320 nm (**b**), 400 nm (**c**), and 500 nm (**d**).

Figure 6 shows the photocatalytic hydrogen production via water splitting of the different catalysts under irradiation at 280 nm (Figure 6a), 320 nm (Figure 6b), 400 nm (Figure 6c), and 500 nm (Figure 6d). The maximum hydrogen production of the unmodified ZnO catalysts was 442 μmol/hg and was obtained with ZnO NWs by irradiation at 280 nm. This high hydrogen production from ZnO NWs was not expected, especially when compared to the maximum hydrogen production of ZnO NPs (86 μmol/hg) and the commercial ZnO (94 μmol/hg). According to a study by Zhang et al. [16], one-dimensional nanostructures, such as nanowires, can enhance the photocatalytic activity due to their large surface-to-volume ratio as compared to other morphologies. Furthermore, ZnO is considered a promising material for solar cells due to the fast electron transport, with reduced recombination loss, and its ease of crystallization [16].

Under irradiation at 320 nm, the hydrogen production of ZnO NWs (365 μmol/hg) decreased when compared to that obtained at 280 nm, but then increased again (427 μmol/hg) at 400 nm. This was not expected either since the wide band gap energy of ZnO (3.37 eV for wurtzite) does not favor the production of hydrogen under visible light. Different studies [16,43,44] have found that surface defects and oxygen vacancies in photocatalysts can play a significant role in their photocatalytic activity. Crystalline defects in ZnO nanowires exist primordially due to oxygen vacancies. Even more, these studies have found that nanoparticles with crystalline defects can exhibit visible light activity even without doping them with transition metals.

Both ZnO NPs and the commercial ZnO obtained similar results in all the wavelengths that were evaluated. At 500 nm, the hydrogen production of the unmodified catalysts was almost zero, with the exception of ZnO NWs that obtained a high value of 350 μmol/hg, showing high catalytic activity. Incorporation of gold and graphene greatly increased hydrogen production in both the UV and visible regions of all the composites. The presence of co-catalysts such as Au and graphene improve the charge separation and suppresses the recombination of excited photogenerated carriers, resulting in a better evolution of H_2_ [26]. Different studies [45,46,47] have demonstrated that when semiconductors, such as ZnO, are doped with noble metal or metal ions, they exhibit a negative shift in the Fermi level that implies a greater degree of electron accumulation in Au-loaded. Thus, such a shift in the Fermi level improves the composite system and enhances the efficiency of the interfacial charge-transfer process. These improvements are in turn associated with a considerable enhancement of the electric near-field [45]. This activity relates strongly to the size and shape-dependent surface charge oscillation known as surface plasmon resonance (SPR) in the presence of light irradiation [26]. Furthermore, the incorporation of graphene on semiconductors creates the p–n junction, which also improves the separation of photogenerated charges [26,48]. The photogenerated holes that were created are then scavenged by the sacrificial agent (S^2–^/SO_3_^2–^), and the electrons are excited to the conduction band. Electrons transferred from the conduction band of the semiconductor are injected into the graphene because graphene has a slightly lower redox potential than the semiconductor conduction band [26,48]. Graphene has a high charge carrier transfer and mobility as a result of its π-conjugated structure, and hence Au nanoparticles dispersed on the graphene can also accept electrons and act as active sites to react with adsorbed H^+^ ions for H_2_ evolution [26,48].

According to other authors [37,49], some conduction electrons can be transferred directly to the Au NPs deposited on the surface of the semiconductor by ohmic interconnection or to carbon atoms on the graphene, and the electrons then react with the adsorbed H^+^ ions to form H_2_. Thus, the synergetic effect between both co-catalysts, plasmonic Au nanoparticles and graphene, can effectively suppress photogenerated charge recombination, enlarge the active adsorption sites and reaction space, and consequently enhance the photocatalytic activity for H_2_ evolution [37,49]. In this regard, Wang et al. [49] reported that Au@TiO_2_–graphene composites had significantly increased the visible light absorption and enhanced the photocatalytic H_2_ production activity compared to the Au@TiO_2_. Luo et al. [37] found that by combining graphene and gold nanoparticles on TiO_2_-P25, the hydrogen production via water splitting increased nine times more than bare TiO_2_-P25.

In this research, the highest hydrogen production of the Au@commercial ZnO–graphene catalysts was 709 μmol/hg and was obtained by 10%Au@ZnO commercial graphene under irradiation at 400 nm. This enhancement represents a difference of 615 μmol/hg when compared to the highest amount obtained by the unmodified commercial ZnO catalyst (94 μmol/hg), and the fact that the maximum production of the commercial catalyst was obtained at 400 nm (visible light) is an indication that the Au NPs are allowing the use of visible light [18,30,37]. On the other hand, appropriate visible light irradiation can induce the SPR effect on the gold nanoparticles and greatly enhance the electron capture capacity [37]. Both reasons affect the generation and separation of charges in photocatalysis, which results in the improvement of photocatalytic properties. The highest amount of hydrogen obtained with 1%Au@commercial ZnO–graphene, 3%Au@commercial ZnO–graphene, and 5%Au@commercial ZnO–graphene catalysts was 405 μmol/hg, 529 μmol/hg, and 589 μmol/hg, respectively, under irradiation at 400 nm.

In the case of Au@ZnO NPs–graphene catalysts, the highest hydrogen production measured was 759 μmol/hg, representing a difference of 673 μmol/hg when compared to the maximum hydrogen production of the unmodified ZnO NPs catalyst (86 μmol/hg), and was obtained with the 10%Au@ZnO NPs–graphene catalyst at 400 nm. The highest hydrogen production for 1%Au@ZnO NPs–graphene, 3%Au@ZnO NPs–graphene, and 5%Au@ZnO NPs–graphene catalysts was 537 μmol/hg, 622 μmol/hg, and 728 μmol/hg, respectively. These results confirm once again that the presence of Au NPs allows the use of visible light to produce hydrogen.

Au@ZnO NWs–graphene catalysts showed the highest hydrogen production (1127 μmol/hg) with a gold loading of 10 wt.% at 400 nm, representing a difference of 685 μmol/hg when compared to the unmodified ZnO NWs catalyst (442 μmol/hg). The highest amount of hydrogen produced at 400 nm with the 1%Au@ZnO NWs–graphene, 3%Au@ZnO NWs–graphene, and 5%Au@ZnO NWs–graphene catalysts was 701 μmol/hg, 828 μmol/hg, and 944 μmol/hg, respectively.

Under irradiation at 500 nm (Figure 6d), the maximum hydrogen production of the Au@commercial ZnO–graphene, Au@ZnO NPs–graphene, and Au@ZnO NWs–graphene catalysts was 628 μmol/hg, 735 μmol/hg, and 1079 μmol/hg, respectively, with a gold loading of 10 wt.%. These high hydrogen productions under low energy irradiation are an indication of the high photocatalytic activity of the composites, especially considering the high band-gap energy (3.37 eV) of ZnO. At wavelengths above 400 nm, the water splitting depends mainly on the Au NPs, due to lack of energy to promote electrons from the valence band to the conduction band of ZnO.

In all cases, the highest amounts of hydrogen reported in this investigation were obtained with the catalysts with the highest surface area (65 m^2^ g^−1^ for 10%Au@ commercial ZnO–graphene; 117 m^2^ g^−1^ for 10%Au@ZnO NPs–graphene; 247 m^2^ g^−1^ for 10%Au@ZnO NWs–graphene). Materials with high surface areas can be attained either by fabricating small particles or clusters where the surface-to-volume ratio of each particle is high, or by creating materials where the void surface area (pores) is high compared to the amount of bulk support material [50]. Multiple studies have demonstrated that the synthesis of high surface area catalysts lead to an increment in the hydrogen production due to the availability of more sites for the interaction of the water molecule with the catalyst [49,50]. In our research, this increase in surface area is primarily achieved by incorporating Au NPs and graphene on the surface of semiconductors.

**Figure 7:**
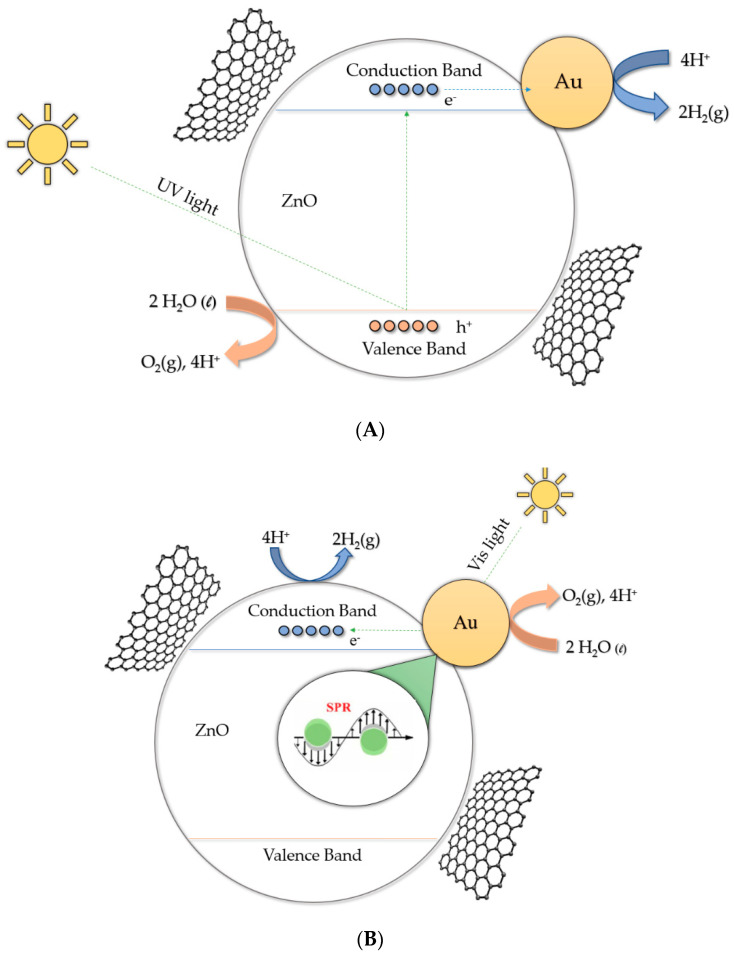
Possible mechanism of hydrogen production under ultraviolet (**A**) and visible (**B**) irradiation for the Au@ZnO–graphene systems.

In the case of Au/graphene–TiO_2_, over the years different possible mechanisms have been proposed for the production of hydrogen by water splitting (see Figure 7). One of the most widely accepted is that when compounds are irradiated with UV light (Figure 7A), a direct photoexcitation of TiO_2_ with photons with energy larger than the bandgap (λ < 380 nm) leads to the generation of electrons in the conduction band, and electron holes in the valence band of the semiconductor [51]. The electron in the conduction band will move to the Au NPs, acting as electron buffers and catalytic sites for hydrogen generation [52]. When irradiated with visible light (λ > 500 nm) photoexcitation of Au NPs occurs, and electrons from the Au NPs are injected into the TiO_2_ conduction band leading to the generation of holes in the Au NPs and electrons in the TiO_2_ conduction band [51,52]. Then, the water molecule gains the electrons in the conduction band and hydrogen is produced. Evidence of the proposed mechanism is the fact that the photocatalytic response for hydrogen generation is consistent with the absorption of the Au surface plasmon band. The incorporation of graphene creates a p–n junction, which improves the separation of photogenerated charges, and the electrons are excited to the conduction band [26]. The electrons transferred from the conduction band of TiO_2_ are injected into the reduced graphene in a graphene/TiO_2_ system because the graphene/graphene redox potential is slightly lower than the CB of TiO_2_ [26]. In addition, some conduction electrons of TiO_2_ likely transfer directly to the Au NPs deposited on the surface of the semiconductor by ohmic interconnection or to carbon atoms on the graphene sheets, and the electrons then react with the adsorbed H^+^ ions to form H_2_ [25,26]. This creates a synergistic effect between both co-catalysts, and they can effectively suppress photogenerated charge recombination, enlarge the active adsorption sites, and consequently enhance the photocatalytic activity [16,25,26]. When irradiated with visible light (λ > 500 nm) (Figure 7B) photoexcitation of Au NPs occurs, and electrons from the Au are injected into the ZnO conduction band, leading to the generation of holes in the Au NPs and electrons in the ZnO conduction band [18]. The water molecule gains the electrons in the conduction band and hydrogen is produced. This proposed mechanism is an oversimplification since different studies [18,19,20] have determined that, due to the gold/semiconductor interfacial contact, the conduction band of the semiconductor undergoes shift toward more negative potentials [18]. Thus, the charge distribution between the Au NPs and the semiconductor causes a shift of the Fermi level toward more negative potentials [18,19].

Table 2 shows the highest amounts of hydrogen obtained with Au@ZnO–graphene catalysts under the evaluated parameters. As already mentioned, and to the best of our knowledge, no results have been reported so far on hydrogen production by water splitting using Au@ZnO–graphene catalysts. Therefore, the results of this research are the first reported on the production of H_2_ using catalysts based on ZnO and graphene–gold. The materials studied in this research will have to be contrasted by other researchers to establish a much deeper knowledge that allows us to know the complex mechanism of hydrogen production with ternary compounds based on ZnO.

## 4. Conclusions

Graphene and different amounts of gold nanoparticles were incorporated on the surface of synthesized ZnO supports (ZnO NWs, ZnO NPs catalysts), and on the commercial form (commercial ZnO). These catalysts were fully characterized by different techniques, and their photocatalytic activity was determined by measuring the hydrogen produced by water splitting under UV–vis irradiation.

The highest amount of the unmodified ZnO support was 442 μmol/hg and was obtained by the ZnO NWs catalyst under irradiation at 280 nm. This unexpectedly high hydrogen production may be attributed to the morphology (nanowires) and possible defects in the crystalline structure. The maximum hydrogen production for the ZnO NPs and commercial ZnO catalysts was 94 μmol/hg and 86 μmol/hg, respectively, at 280 nm.

The maximum hydrogen production obtained with the commercial ZnO composites containing gold and graphene was 709 μmol/hg at 400 nm and was obtained with a gold loading of 10 wt.%. The enhancement in the hydrogen production was 7.5 times higher than that reported by the commercial ZnO.

The higher hydrogen production for the Au@ZnO NPs–graphene catalysts was 759 μmol/hg at 400 nm and was obtained with 10%Au@ZnO NPs–graphene. The enhancement in the hydrogen production was 8.8 times higher than that reported by the ZnO NPs catalyst.

In the case of the Au@ZnO NWs–graphene composites, the higher hydrogen production (1127 μmol/hg) was obtained with the 10%Au@ZnO NWs–graphene under irradiation at 400 nm.

The catalysts did not show a reduction in the surface area nor in the hydrogen production with the increment in gold loadings and incorporation of graphene. These results suggest that the best graphene and gold loading for the Au@ZnO–graphene catalysts could be higher than 1 and 10 wt.%, respectively.

## Figures and Tables

**Table 1:** BET surface area of the Au@ZnO–graphene composites.

	Commercial ZnO (m^2^ g^−1^)	ZnO NPs (m^2^ g^−1^)	ZnO NWs (m^2^ g^−1^)
**Unmodified**	18	67	167
1%Au–Graphene *	48	96	201
3%Au–Graphene *	51	103	212
5%Au–Graphene *	56	109	223
10%Au–Graphene *	65	117	247

* The amount of graphene in all the catalysts was 1 wt.%.

**Table 2:** Highest amounts of hydrogen production via water splitting obtained with Au@ZnO–graphene catalysts under UV–vis light.

Author	H_2_ Production (μmol)	Source (nm)	Irradiation Time (h)	ZnO Crystal Structure *	Reaction Mixture	Au (wt.%)	Graphene (wt.%)
**This work ZnO commercial**	709	200 > λ > 400	2	W	Water: 0.5 M Na_2_S, 0.03 M Na_2_SO_3_	10	1
**This work ZnO NPs**	759	200 > λ > 400	2	W	Water: 0.5 M Na_2_S, 0.03 M Na_2_SO_3_	10	1
**This work ZnO NWs**	1127	200 > λ > 700	2	W	Water: 0.5 M Na_2_S, 0.03 M Na_2_SO_3_	10	1

* W = Wurtzite.
